# Lipocalin 2 Regulates Inflammation during Pulmonary Mycobacterial Infections

**DOI:** 10.1371/journal.pone.0050052

**Published:** 2012-11-20

**Authors:** Lokesh Guglani, Radha Gopal, Javier Rangel-Moreno, Beth Fallert Junecko, Yinyao Lin, Thorsten Berger, Tak W. Mak, John F. Alcorn, Troy D. Randall, Todd A. Reinhart, Yvonne R. Chan, Shabaana A. Khader

**Affiliations:** 1 Division of Pulmonary Medicine, Department of Pediatrics, University of Pittsburgh School of Medicine, Pittsburgh, Pennsylvania, United States of America; 2 Division of Infectious Diseases, Department of Pediatrics, University of Pittsburgh School of Medicine, Pittsburgh, Pennsylvania, United States of America; 3 Division of Allergy, Immunology and Rheumatology, Department of Medicine, University of Rochester Medical Center, Rochester, New York, United States of America; 4 Department of Infectious Diseases and Microbiology, Graduate School of Public Health, University of Pittsburgh, Pittsburgh, Pennsylvania, United States of America; 5 The Campbell Family Institute for Breast Cancer Research and the Ontario Cancer Institute, University Health Network, Toronto, Canada; 6 Division of Pulmonary, Allergy and Critical Care Medicine, Department of Medicine, University of Pittsburgh School of Medicine, Pittsburgh, Pennsylvania, United States of America; French National Centre for Scientific Research, France

## Abstract

Pulmonary tuberculosis (TB), caused by the intracellular bacteria *Mycobacterium tuberculosis*, is a worldwide disease that continues to kill more than 1.5 million people every year worldwide. The accumulation of lymphocytes mediates the formation of the tubercle granuloma in the lung and is crucial for host protection against *M.tuberculosis* infection. However, paradoxically the tubercle granuloma is also the basis for the immunopathology associated with the disease and very little is known about the regulatory mechanisms that constrain the inflammation associated with the granulomas. Lipocalin 2 (Lcn2) is a member of the lipocalin family of proteins and binds to bacterial siderophores thereby sequestering iron required for bacterial growth. Thus far, it is not known whether Lcn2 plays a role in the inflammatory response to mycobacterial pulmonary infections. In the present study, using models of acute and chronic mycobacterial pulmonary infections, we reveal a novel role for Lcn2 in constraining T cell lymphocytic accumulation and inflammation by inhibiting inflammatory chemokines, such as CXCL9. In contrast, Lcn2 promotes neutrophil recruitment during mycobacterial pulmonary infection, by inducing G-CSF and KC in alveolar macrophages. Importantly, despite a common role for Lcn2 in regulating chemokines during mycobacterial pulmonary infections, Lcn2 deficient mice are more susceptible to acute *M.bovis* BCG, but not low dose *M.tuberculosis* pulmonary infection.

## Introduction

Pulmonary tuberculosis (TB), caused by the intracellular bacteria *Mycobacterium tuberculosis*, is a widespread disease that continues to kills more than 1.5 million people every year worldwide. Furthermore, approximately one-third of the world’s population is infected with *M.tuberculosis* and about 5–10% of these individuals will develop clinical disease over their lifetime. Induction of inflammatory molecules in response to infection mediates cellular accumulation, formation of the tubercle granuloma and host protection against *M.tuberculosis* infection. However, the generation of the tubercle granuloma is also thought to be the basis for the immunopathology associated with the disease. Although it is known that the granuloma is dependent on the recruitment and localization of lymphocytes [1] and that both inflammatory chemokines [2], [3], [4], [5] and homeostatic chemokines [1], are involved in cellular accumulation, very little is known about the regulatory mechanisms that restrain chemokines and regulate inflammation associated with TB granuloma formation.

Lipocalin 2 (Lcn2), also known as neutrophil gelatinase-associated lipocalin, siderocalin, 24p3 or uterocalin is a member of the lipocalin family of proteins and possesses a barrel shaped tertiary structure with a hydrophobic pocket that can bind lipophilic molecules [6]. The best-characterized role of Lcn2 is its ability to bind to siderophores that are made by pathogenic bacteria, such as *Escherichia coli*, and its resultant ability to restrict bacterial growth [7], [8], [9]. Accordingly, Lcn2 gene deficient mice (Lcn2KO) exhibit higher mortality rates than control mice when challenged with pathogenic *E.coli* [7], [8], [9]. Furthermore, Lcn2 is an acute phase protein, as it is induced under inflammatory conditions [7], [10] and is implicated in control of cell growth and differentiation, tissue involution and apoptosis [7], [11], [12]. Accordingly, high Lcn2 levels have been associated with malignancy in various cancers [13], [14], [15].

Recently it has been documented that Lcn2 can also bind to soluble siderophores of mycobacteria, namely carboxymycobactins [16], and that Lcn2 can inhibit the *in vitro* growth of *M.tuberculosis* [17], [18] as well as the intracellular replication of *M.tuberculosis* in cultured macrophage cell lines [19]. Furthermore, Lcn2 can also inhibit the growth of attenuated mycobacterium strain, *M.bovis* Bacille Calmette Guerin (*M.bovis* BCG), in cultured murine alveolar epithelial cells [18]. In addition, in response to acute intratracheal high dose infection with virulent *M.tuberculosis* H37Rv, mice deficient in Lcn2 are more susceptible than wild type (WT) infected mice [18]. However, it is not known whether Lcn2 plays a role following a more physiological exposure to *M.tuberculosis,* such as pulmonary infection with a low dose of aerosolized *M.tuberculosis*. Moreover, since the expression of Lcn2 is limited to mucosal tissues, such as the trachea, lung, stomach, salivary gland and colon [20], it is likely that Lcn2 plays an inflammatory role at mucosal surfaces. In this study, we reveal a new role for Lcn2 in regulating lung inflammation and T cell accumulation during mycobacterial pulmonary infections, by restraining CXCL9 induction. Accordingly, absence of Lcn2 results in enhanced induction of lung CXCL9 mRNA and increased inflammation following both acute *M.bovis* BCG pulmonary infection and low dose chronic pulmonary *M.tuberculosis* infection. Importantly, despite a common role for Lcn2 in regulating CXCL9 expression during mycobacterial pulmonary infections, Lcn2KO mice are more susceptible to acute *M.bovis* BCG pulmonary infection, but not low dose *M.tuberculosis* pulmonary infection.

## Materials and Methods

### Animals

C57BL/6 (WT) mice were purchased from Taconic Farms, Inc. (Hudson, NY) and Lcn2KO mice [21] were obtained from Dr. Tak W Mak, University Health Network, Toronto, Canada and were bred and maintained at Taconic Farms. The mice used for all the experiments were age- and sex-matched and were infected between the ages of 6 to 8 weeks in accordance with the University of Pittsburgh IACUC guidelines under protocol 1106203.

### Experimental *M.tuberculosis* and *M.bovis* BCG Infection

*M.tuberculosis* H37Rv strain or *M.bovis* BCG was grown in Proskauer Beck medium containing 0.05% Tween 80 to mid-log phase and frozen in 1 ml aliquots at –70°C. For aerosol infections with *M.tuberculosis*, the animals were infected with ∼100 bacteria using a Glas-Col (Terre Haute, IN) airborne infection system as described before [22]. In some experiments, mice were intratracheally infected with 5×10^6^ *M.bovis* BCG in 50 µl of PBS as reported before [23]. All experiments included 4–5 mice and were repeated atleast twice independently.

### Determination of Bacterial Load

Mice were euthanized by CO_2_ asphyxiation, and the organs were excised and individually homogenized in 0.9% physiological saline, and serial dilutions of the organ homogenates were plated on nutrient 7H11 agar. Bacterial colony formation was counted after 3 wk of incubation at 37°C and Log 10 CFU per organ calculated.

### Preparation of Single Cell Suspensions

Lung cell suspensions were prepared by perfusing cold heparinized saline injected through the right side of the heart of the dissected animals until the lungs appeared white [24]. The lobes of the lungs were removed and placed in ice-cold DMEM (Mediatech-Cellgro) followed by sectioning using sterile razor blades. To digest the collagen matrix, the lung tissue samples were then incubated in DMEM containing collagenase IX (0.7 mg/ml; Sigma-Aldrich) and DNase (30µg/ml; Sigma-Aldrich) at 37°C for 30 min [22]. Following this treatment, the digested lung tissue was gently passed through a 70-µm pore size nylon tissue strainer (Falcon; BD Biosciences). The resultant single-cell suspension of all organs was treated with Gey’s solution to remove any residual RBCs, washed twice, and counted. Cells from this single cell suspension were used for flow cytometry analyses.

### Immunohistochemistry and Morphometric Analysis

Lung lobes were instilled with 10% neutral buffered formalin and embedded in paraffin. Lung sections were stained with hematoxylin and eosin and inflammatory features were evaluated by light microscopy (Research Histology Core, University of Pittsburgh). For immunofluorescent staining, formalin-fixed, lung sections were cut, immersed in xylene to remove paraffin and then hydrated in alcohol, 96% alcohol and PBS. Antigens were unmasked with a DakoCytomation Target Retrieval Solution and non-specific binding was blocked with 5% (v/v) normal donkey serum and Fc block (BD Pharmingen, San Diego, CA). Endogenous biotin (Sigma Aldrich) was neutralized by adding first avidin, followed by incubation with biotin. Sections were probed with Biotinylated rat anti Gr-1 (BD Pharmingen) to detect neutrophils, anti B220 to detect B cells (Clone RA3-6B2, BD Pharmingen. San Diego, CA) and anti CD3 to detect T cells (Clone M-20, Santa Cruz Biotechnology. Santa Cruz, CA) in the inflammatory lesions. Primary antibodies were visualized with a secondary antibody, donkey-anti-rat, conjugated to Alexa-Fluor 488 (Invitrogen, Carlsbad, CA). Slow fade gold antifade with DAPI (Molecular probes, Eugene, OR) was used to counterstain tissues and to detect nuclei. Images were obtained with a Zeiss Axioplan 2 microscope and were recorded with a Zeiss AxioCam digital camera. Caudal lobes underwent morphometric analysis in a blinded manner. The area occupied by cellular infiltrates was determined with the automated, morphometric tool of the Zeiss Axioplan microscope (Zeiss), which determines the area defined by the squared micron value for each granuloma measured, as previously described by us [1], [25].

### In Situ Hybridization

Mouse Lcn2 cDNA was RT-PCR amplified with primers BF.mLcn2_F1 (5′- ACCTAGTAGCTGTGGAAACC-3′) and BF.mLcn2_R1 (5′-TCAGCCACA CTCACCACCCA-3′) using reverse transcribed total RNA from cultured murine splenocytes. PCR products were ligated to the pGEM-T vector (Promega) and DNA sequenced. The pGEMT-Lcn2 plasmid was linearized by restriction digest. Gene-specific riboprobes were synthesized by *in vitro* transcription using a Maxiscript SP6/T7 kit (Ambion) and unincorporated nucleotides were removed using RNA Mini Quick Spin Columns (Roche). Paraffin embedded tissue specimens were pretreated as described [26], following deparaffinization in xylenes and rinsing in ethanol. *In situ* hybridization with ^35^S-labeled riboprobes against Lcn2 was performed at 50^o^C overnight with 0.1M dithiothreitol included in the hybridization mix as described [27]. Tissue sections were coated with NTB-2 emulsion (Kodak) and exposed at 10^o^C for 7 days. The sections were counterstained with Hematoxylin (Vector) and mounted with Permount (Fisher). Microscopic fields were visualized using a Olympus BX41 microscope and captured using a SPOT RT3 digital camera (Diagnostics Instruments, Inc). CXCL9 mRNA was detected as previously described [26].

### Flow Cytometry

Single cell suspensions were stained with fluorochrome-labeled antibodies specific for CD3 (17A2), Gr1 (RB6-8C5) and CD11b (M1/70). For intracellular staining, cells stimulated with Phorbol myristate acetate (50ng/ml), ionomycin (750 ng/ml; Sigma Aldrich) and Golgistop (BD Pharmingen), were surface stained, permeabilized with Cytofix/Cytoperm solution (BD Pharmingen) and stained with anti-IFNγ (XMG1.2).

### Real Time PCR (RT-PCR)

Lung tissue from infected and control mice was homogenized and frozen in RLT buffer (Qiagen Inc, Valencia CA). Extraction of RNA was done as per protocol, followed by reverse transcription and amplification using FAM-labeled probe and primers on the ABI Prism 7400HT detection system (Applied Biosystems, Foster City CA). Calculation of ΔΔct was done from the fold increase in signal from infected samples over that of uninfected samples. The primer and probes sequences have been previously published [1], [24] or were purchased (Applied Biosystems, Foster City CA).

### Isolation of Alveolar Macrophages, Fibroblasts and Tracheal Epithelial Cells

Alveolar Macrophages were obtained from mice by performing bronchoalveolar lavage with 0.2mM EDTA solution. Mice were dissected to expose their trachea and cannulated with a blunt end 18 gauge needle. Six lavages were performed per mouse with 1ml volume per lavage. The cells were all pooled, spun, resuspended in Minimum Essential Media (MEM, Gibco #11095-080) with 1% Penicillin-Streptomycin, and 10% Fetal Bovine Serum, counted and plated in 96 well plates which were incubated for 1 hour at 37°C, following which the wells were washed to remove non-adherent cells. The adherent alveolar macrophages were then rested overnight before being used in experiments.

Mouse tracheal epithelial cells (MTEC) were generated using the air liquid interface as described before [26]. Mouse tracheas were placed in 0.15% Pronase solution, tracheal cells removed with DNase, followed by incubation in MTEC Basic media with 10% FBS. The cells in supernatant were resuspended in MTEC Plus media and then added to collagen coated transwell membranes (Corning Costar #3460), followed by change of media on days 3, 5 and 6 in both apical and basal compartments. The resistance of cells to electric current was checked on days 5, 6 and 7 and cells were switched to air-liquid interface once a resistance of 800–1000 ohms was reached. At this point, media was removed from apical compartment and media from basal compartment was replaced with 2% NuSerum (BD Biosciences #355100) with Retinoic Acid (Sigma R-2625). For generation of lung fibroblast, lungs were perfused with 5 units/ml dispase followed by 1% low melting agarose, washed in PBS and incubated in dispase for 30 minutes at 37^o^C. Single cell suspensions were then obtained from DNase/Collagenase treated lungs and fibroblasts generated by passaging 2–4 times. Recombinant Lcn2 was made as described [21] and used at a concentration of 10 ug/ml for all *in vitro* experiments. The endotoxin levels were tested in preparations of recombinant Lcn2 and found to be low (12 ng/ml total protein preparation).

### Determination of Protein Levels

Protein levels of cytokines and chemokines were measured in cell culture supernatants or lung homogenates using a mouse Luminex assay (Linco**/**Millipore). Lipocalin 2 protein was measured as previously described [21]. Briefly, Ab sandwich ELISA was performed per standard protocol by coating 96-well plates with affinity-purified anti-mouse lipocalin 2, application of 100 µl of diluted protein lysate, detection with monoclonal rat anti-mouse lipocalin 2 (R&D Systems), and HRP-conjugated goat anti-rat IgG (Invitrogen), followed by colorimetric development using a 3,3′,5,5′-tetramethylbenzidine substrate reagent set (BD Biosciences).

### Statistical Analysis

Comparison of the means between the uninfected and infected sample groups was performed with the two tailed Student’s *t*-test using GraphPad Prism 5 software (La Jolla, CA). Differences were considered significant at *p*≤0.05. The data from bacterial growth and RT-PCR were log-transformed for statistical analyses.

## Results

### Lcn2 Expression is Induced in the Lungs in Response to Low Dose Aerosol *M.tuberculosis* Infection

**Figure 1:**
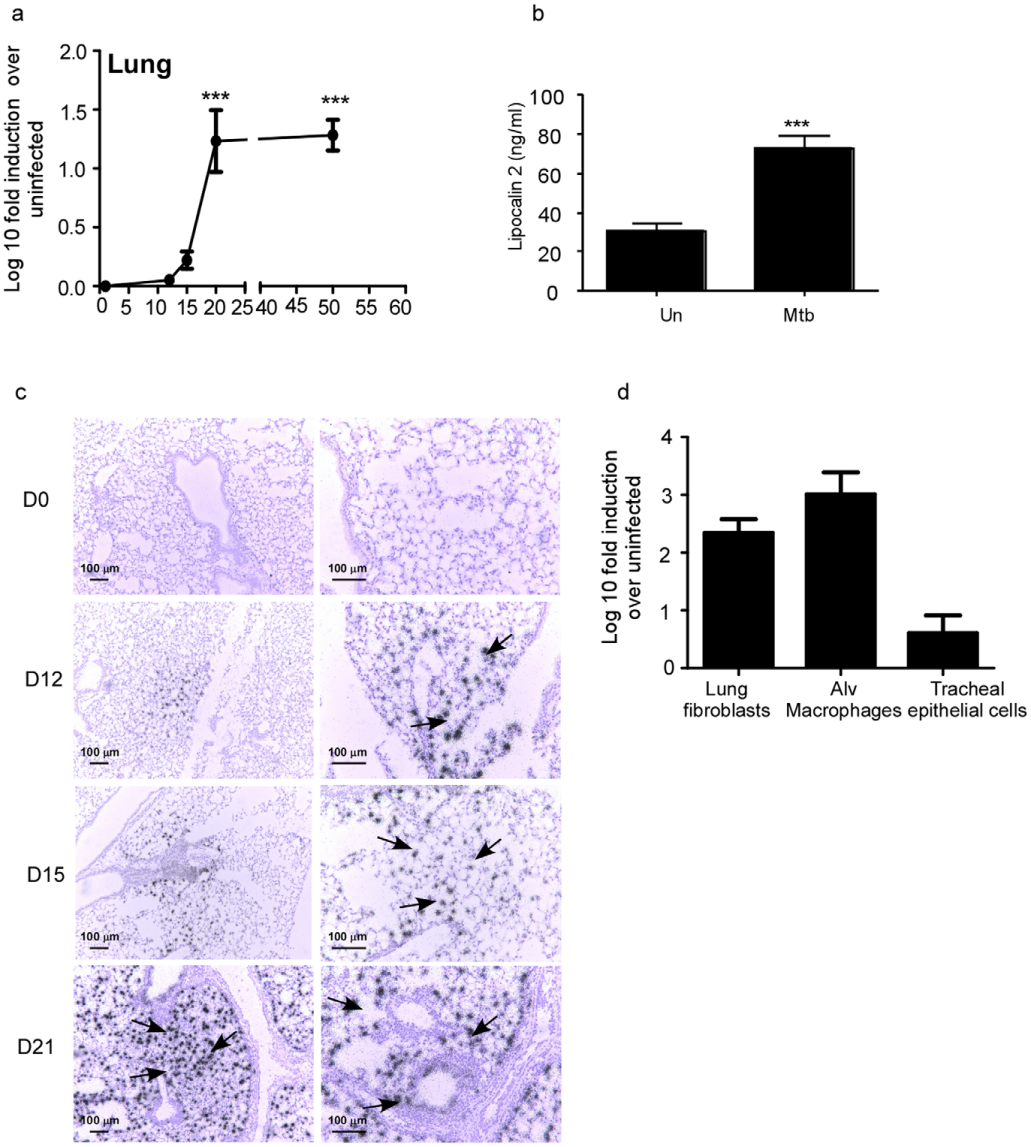
Lcn2 is induced in murine lungs in response to low dose aerosolized *M.tuberculosis* infection. Wild type (WT) mice were infected with ∼100 CFU *M.tuberculosis* via the aerosol route and at specific times after infection, lung tissue was harvested and processed to extract RNA and the expression of Lcn2 mRNA was determined by RT-PCR. Log 10 fold induction of Lcn2 gene in the lungs of *M.tuberculosis*-infected mice over lungs from uninfected controls is shown (a). WT *M.tuberculosis*-infected lungs (Mtb) on day 30 post infection or uninfected lungs (Un) were assayed for Lcn2 protein by ELISA (b). In situ hybridization with a Lcn2 cRNA probe was carried out in uninfected and *M.tuberculosis*-infected lung samples at various time points post infection (c). Original magnification. 100X-left panel; 200X-rightpanel). Lung fibroblasts, alveolar macrophages and tracheal epithelial cells were generated from WT lungs and cells were exposed to irradiated *M.tuberculosis* (100 µg/ml) for 48 hours. The induction of Lcn2 mRNA was measured by RT-PCR and the log 10 fold induction was measured in treated samples relative to untreated controls (d). The data points represent the mean and SD for four mice for each time point and one experiment representative of two shown. _***_, *p* ≤0.0005.

Lcn2 gene expression is induced in the lung in response to *M.bovis* BCG acute pulmonary infection [18]. However, *M.bovis* BCG is an attenuated strain of mycobacterium and it is not known whether Lcn2 is induced in the lung in response to exposure to pathogenic mycobacterium such as *M.tuberculosis* H37Rv. Therefore, we infected wild type mice with low dose aerosolized *M.tuberculosis* H37Rv and found that Lcn2 mRNA was induced between day 15 and day 21 post-*M.tuberculosis* infection and the mRNA levels were maintained at later time points (Figure 1a). Furthermore, the timing of induction of Lcn2 mRNA between days 15–21 coincided with the induction of other protective host immune genes, such as IFNγ and TNFα, in the lungs of *M.tuberculosis*-infected mice [28]. In addition, we found significant induction of Lcn2 protein in day 30 WT Mtb-infected lungs (Figure 1b).

Epithelial cells and macrophages are considered to be one of the major sources of Lcn2 expression [18], [26]. Interestingly, following *M.tuberculosis* infection, the early induction of Lcn2 mRNA (day 12–15) was detected within the lung parenchyma consistent with type II epithelial cells and macrophages, whereas late expression of Lcn2 mRNA (day 21) was detected both within the lung parenchyma and the airways of the *M.tuberculosis*-infected lungs (Figure 1c). These data suggest that multiple cell types can likely produce Lcn2 in response to *M.tuberculosis* infection. Accordingly, we found that following *M.tuberculosis* exposure, lung fibroblasts and alveolar macrophages produced high levels of Lcn2 mRNA, while Mouse Tracheal Epithelial Cells (MTECs) induced lower levels of Lcn2 mRNA (Figure 1d). IL-23 is a key mediator of IL-17 and IL-22 responses [29], [30], [31], and IL-22 and IL-17 can induce Lcn2 expression in lung epithelial cells and participate in protection against gram-negative bacteria [26]. However, we found that Lcn2 mRNA induction was not significantly different among wild type, IL-23KO, IL-22KO and IL-17AKO *M.tuberculosis*-infected lungs (data not shown). These data together suggest that following low dose pulmonary infection with *M.tuberculosis,* Lcn2 mRNA is induced both within the lung parenchyma and epithelium, and that several lung cell types can produce Lcn2 in response to *M.tuberculosis* exposure.

### Lcn2 is not Required for Protective Immunity Against Primary Low Dose *M.tuberculosis* Aerosol Infection

**Figure 2:**
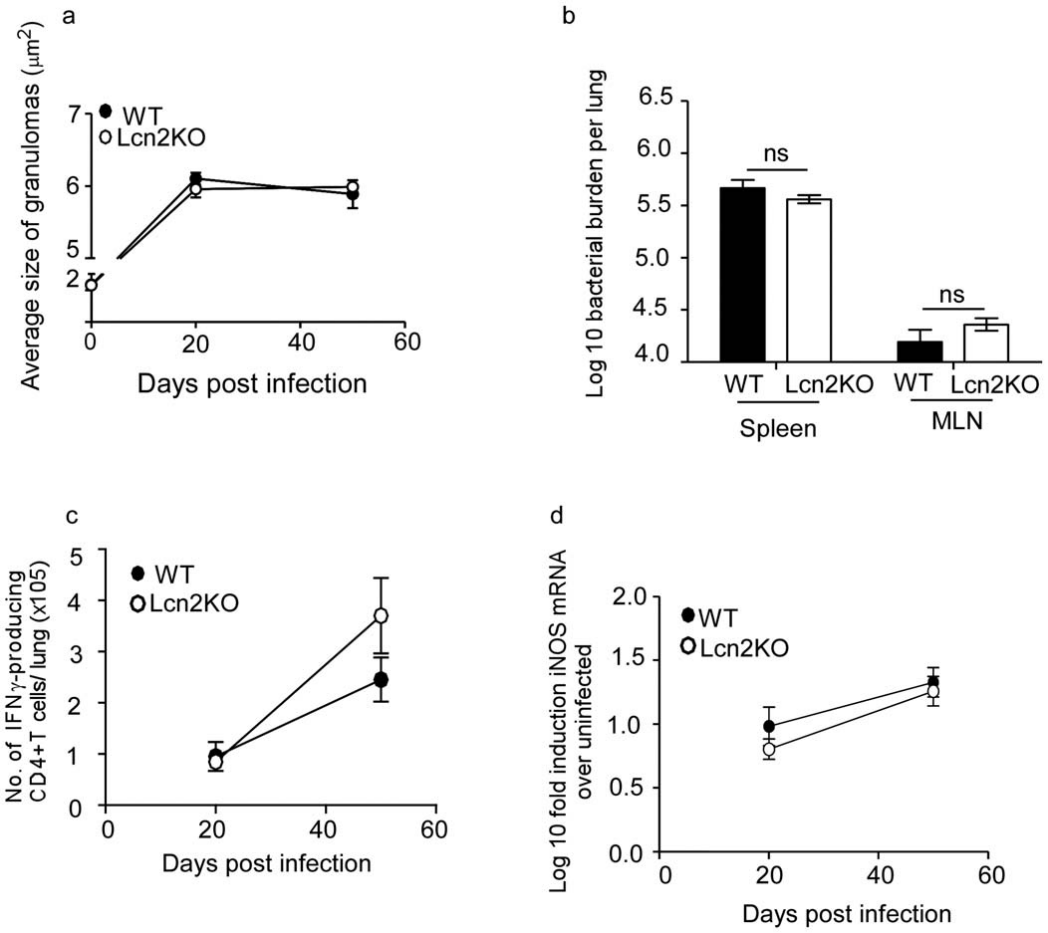
Lcn2 is not required for protective immunity against low dose *M.tuberculosis* infection. WT and Lcn2KO mice were infected with low dose aerosolized *M.tuberculosis* and the bacterial burden determined in the lungs at different time points post infection (a) and spleens and mediastinal lymph nodes (MLN) on day 50 post infection (b). Lymphocytes were isolated from the lung and the number of CD4^+^ IFNγ^+^ producing T cells were determined by intra cellular staining and flow cytometry following stimulation with PMA and Ionomycin (c). Lung from uninfected and infected WT and Lcn2KO mice were processed and the Log 10 induction of iNOS mRNA in infected samples relative to uninfected control samples was determined by RT-PCR (d). The data points represent the mean and SD for four mice for each time point and one experiment representative of two shown. ns-not significant.

To determine whether Lcn2 was required for protective immunity during low dose *M.tuberculosis* infection, we assessed the bacterial burden in *M.tuberculosis*-infected wild type (WT) and Lcn2 knock out (Lcn2KO) mice and found that the total lung bacterial burdens in Lcn2KO and WT mice were similar (Figure 2a). In addition, dissemination to the peripheral organs was also similar, since bacterial burdens in spleen and lymph node were comparable between WT and Lcn2KO mice on day 50 post infection (Figure 2b). These results were consistent with the comparable accumulation of IFNγ producing CD4^+^ T cells (Figure 2c) in *M.tuberculosis*-infected lungs and similar induction of iNOS mRNA (Figure 2d) in WT and Lcn2KO lungs. These data suggest that even in the absence of Lcn2, IFNγ T cell responses are generated effectively to induce macrophage activation that can mediate protective immunity against *M.tuberculosis* infection.

### Lcn2 Regulates Pulmonary Inflammation during TB

**Figure 3:**
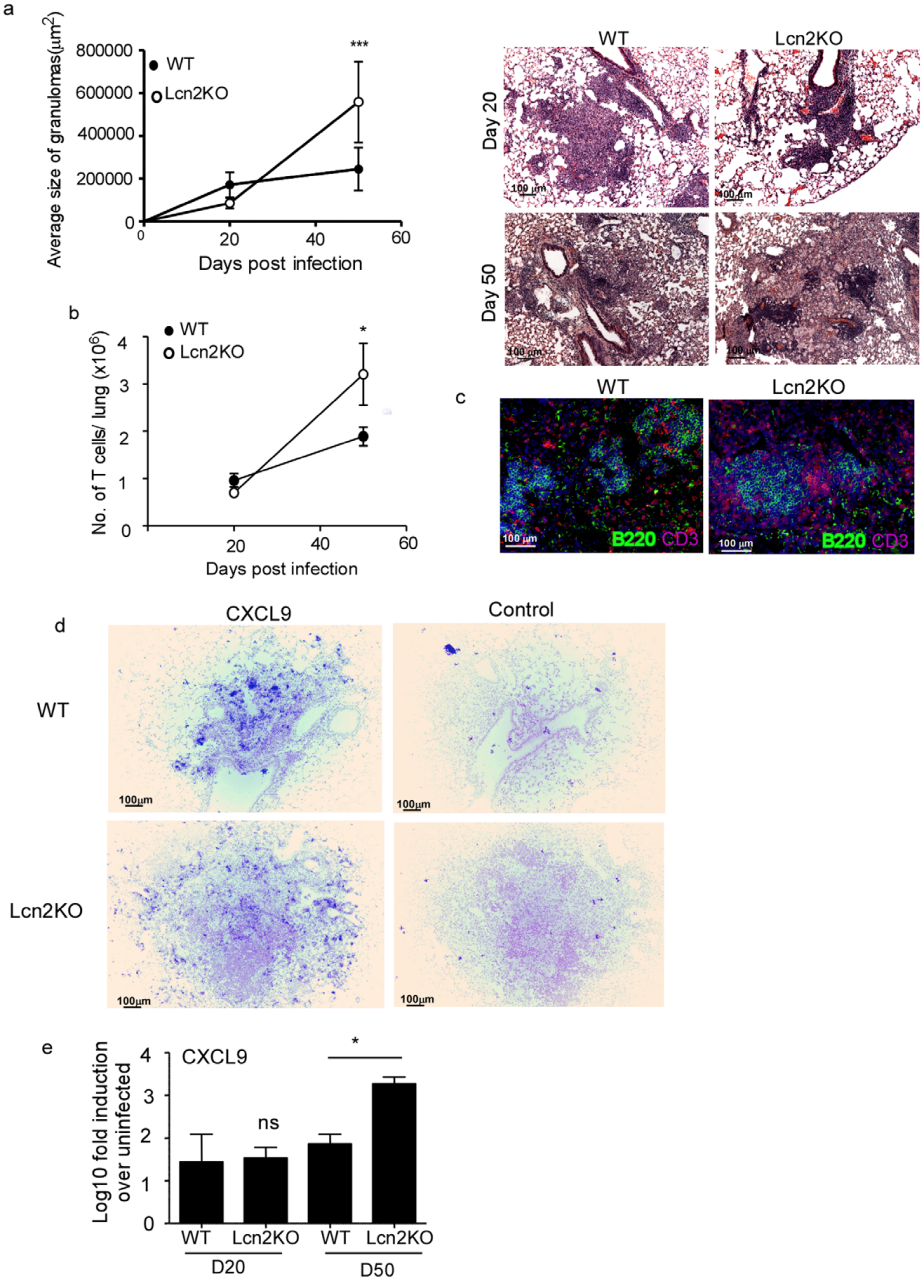
Lcn2 regulates pulmonary inflammation during TB. WT and Lcn2KO mice were infected with low dose aerosolized *M.tuberculosis* and formalin-fixed paraffin-embedded lungs were stained using H&E. Morphometric analysis of the average size of the granuloma in H&E stained sections of *M.tuberculosis*-infected WT and Lcn2KO lungs was determined and a representative section of the lung granuloma is shown for D20 and D50 post *M.tuberculosis* infection (a). Original magnification shown-100X. Lymphocytes were isolated from the lungs of WT and Lcn2KO *M.tuberculosis*-infected mice at day 20 and day 50 post infection and the number of CD3^+^ T cells determined by surface staining and flow cytometry (b). Lung sections from *M.tuberculosis*-infected WT and Lcn2KO mice at D50 post infection were stained for B220 (green) and CD3 (red) and shown at 200X magnification (c). Formalin-fixed, paraffin-embedded lung sections from WT and Lcn2KO from day 50 *M.tuberculosis*-infected mice were assayed for CXCL9 mRNA localization by ISH using a murine CXCL9 mRNA probe or control probe (d) and total lung CXCL9 mRNA levels induced quantitated using RT-PCR in day 20 (D20) or day 50 (D50) *M.tuberculosis-*infected mice (e). Original magnification-100X (d). The data points represent the mean and SD for four mice for each time point and one experiment representative of two shown._ *_, *p* ≤0.05._ ***_, *p* ≤0.0005.

TB is an inflammatory disease of the lung and the formation of the granuloma is a hallmark of the disease. Therefore, despite Lcn2 not being required for overall bacterial control against Mtb infection (Figure 2), we next examined if Lcn2 had a role in granuloma formation. We quantified the average size of the lung granulomas in WT and Lcn2KO *M.tuberculosis*-infected mice by histology. At early stages of the infection, we found that the average size of granulomas were similar in WT and Lcn2KO mice (Figure 3a). However, later during chronic *M.tuberculosis* infection, we found the average size of granulomas were significantly increased in the Lcn2KO mice when compared to granulomas found in lungs of *M.tuberculosis*-infected WT mice (Figure 3a). To further validate the role for Lcn2 in inflammation during *M.tuberculosis* infection, we compared the T cell accumulation in response to pulmonary *M.tuberculosis* infection in WT and Lcn2KO *M.tuberculosis*-infected lungs. Despite similar numbers of lung T cells in WT and Lcn2KO mice at early time points, the T cell numbers were significantly increased in day 50 *M.tuberculosis*-infected Lcn2KO lungs when compared to WT *M.tuberculosis*-infected lungs (Figure 3b). This was consistent with higher accumulation of T cells histologically within granulomas of Lcn2KO *M.tuberculosis*-infected lungs (Figure 3c). These data suggest a novel function for Lcn2 in regulating lymphocytic recruitment and granuloma formation during pulmonary TB.

Inflammatory chemokines such as CXCL9,10 and 11 [2] and CCL2 [3], [4], [5] are critical for lymphocytic recruitment and mediating granuloma formation during TB. Logically, mice deficient in these chemokines or their receptors also have decreased lymphocytic recruitment and decreased granulomatous inflammation when compared to WT mice [2], [3], [4], [5]. Therefore, we hypothesized that Lcn2 may limit expression of some CXCR3-ligating chemokines and restrain lymphocytic accumulation and inflammation during TB. Consistent with this hypothesis, we found increased localization of mRNA for one of the CXCR-3 ligating chemokines, namely CXCL9 mRNA, within granulomas of *M.tuberculosis*-infected Lcn2KO mice when compared to WT *M.tuberculosis*-infected lungs (Figure 3d). In addition, we found that increased inflammation during chronic infection coincided with increased induction of total CXCL-9 mRNA levels in Lcn2KO *M.tuberculosis*-infected lungs when compared to WT infected lungs (Figure 3e). However, we did not find any differences in total CXCL10 mRNA or localization within the granulomas in WT and Lcn2KO *M.tuberculosis*-infected lungs during both early and chronic tuberculosis (data not shown).These novel data suggest that Lcn2 regulates some lymphocyte recruiting chemokines such as CXCL9, to constrain lymphocytic infiltration and granuloma formation during TB.

### Lcn2 Promotes Production of Neutrophil-attracting Chemokines and Neutrophil Recruitment during Pulmonary TB

**Figure 4:**
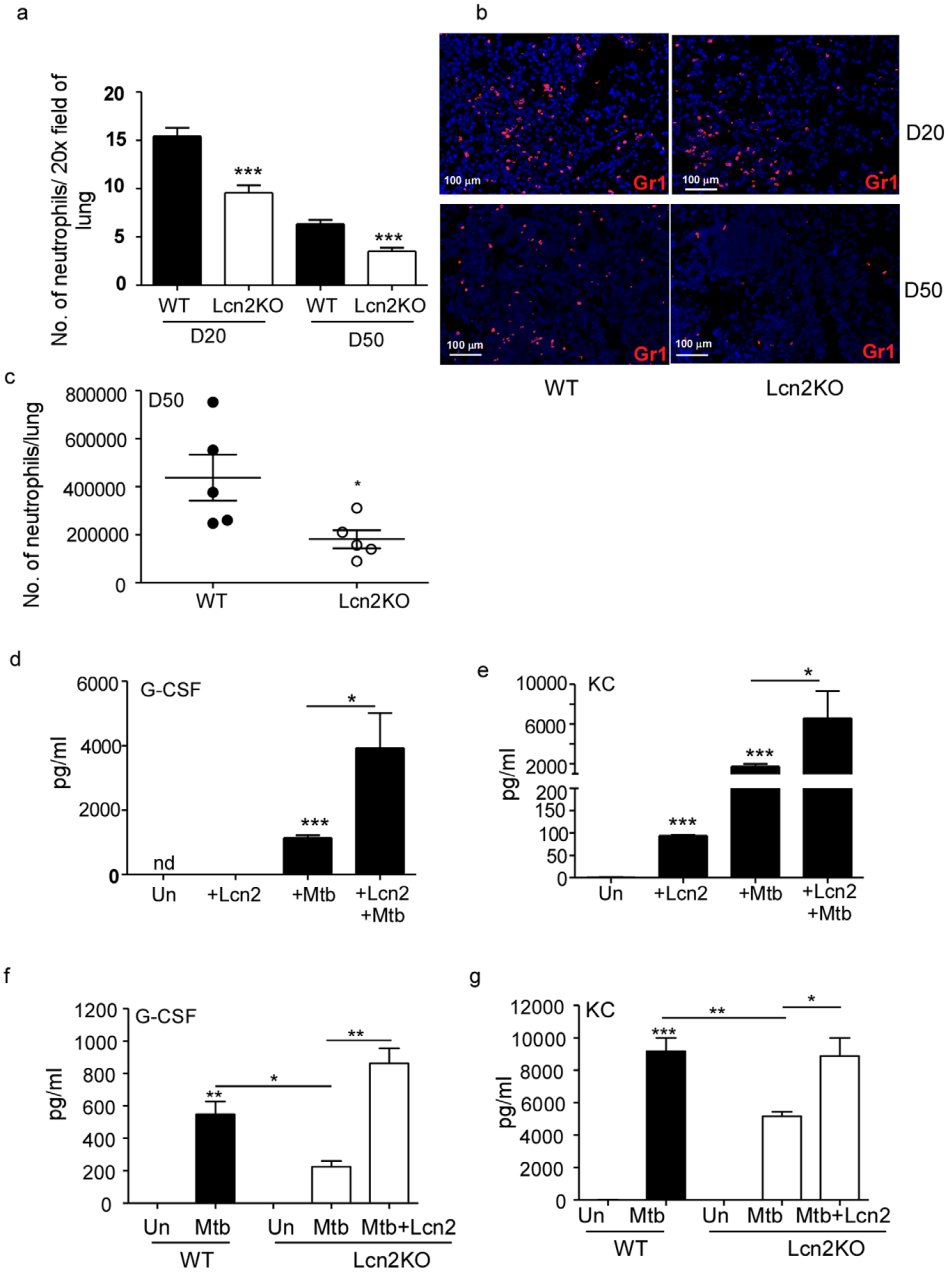
Lcn2 is required for neutrophil recruitment to the lung during TB. WT and Lcn2KO were infected with low dose aerosolized *M.tuberculosis* infection as described in Figure 2. Formalin-fixed paraffin-embedded lung sections from infected WT and Lcn2KO mice were stained for neutrophils (Gr1-red) by immunofluorescence. The number of neutrophils per 20X field was estimated at D20 and D50 (a) and a representative image is shown at 200X magnification (b). Lung cells were isolated from WT and Lcn2KO *M.tuberculosis*-infected lungs at D50 post infection and the total number of neutrophils (Gr1^+^CD11b^+^) determined by surface staining and flow cytometry (c). Alveolar macrophages isolated from WT mice were exposed to *M.tuberculosis* (100 µg/ml) alone, Lcn2 alone (10 µg/ml), or both Lcn2 (10 µg/ml) and *M.tuberculosis* (100 µg/ml) and levels of G-CSF (d), KC (e) were measured in culture supernatants after 48 hours. Alveolar macrophages were isolated from WT or Lcn2KO mice were exposed to *M.tuberculosis* (100 µg/ml) and exogenous Lcn2 (10 µg/ml) and the levels of G-CSF (f) and KC (g) determined in culture supernatant after 48 hours. The data points represent the mean and SD for 3–4 samples for each time point and one experiment representative of two shown._ *_, *p* ≤0.05._ **_, *p* ≤0.005._ ***_, *p* ≤0.0005.

Our data show that Lcn2 has a regulatory role in induction of lymphocyte-attracting chemokines and that absence of Lcn2 during *M.tuberculosis* infection results in enhanced T cell recruitment and increased granuloma inflammation. Paradoxically, we also found, upon immunofluorescent staining for neutrophilic accumulation in the lungs, that Lcn2KO mice had significantly reduced neutrophilic influx compared to WT mice (Figure 4a, b). This also coincided with decreased total numbers of neutrophils in Lcn2KO *M.tuberculosis*-infected lungs compared to WT *M.tuberculosis*-infected lungs (Figure 4c). These data suggest that, apart from its regulatory role in cellular inflammation, Lcn2 may promote neutrophil generation and recruitment. Alveolar macrophages are likely first-responders to infection that would produce these molecules. We show that following exposure to *M.tuberculosis*, alveolar macrophages produced G-CSF, a key regulator of neutrophil production (Figure 4d) and KC, a neutrophil chemoattractant (Figure 4e). Interestingly, Lcn2 treatment alone is sufficient to induce neutrophil chemoattractant, KC in alveolar macrophages (Figure 4e). Addition of exogenous Lcn2 to *M.tuberculosis* exposed alveolar macrophages resulted in significantly higher induction of G-CSF and KC than *M.tuberculosis* alone. Since *M.tuberculosis* exposure induces Lcn2 expression in alveolar macrophages (Figure 1d), we then tested whether the induction of G-CSF and KC was dependent on endogenous Lcn2 expression in alveolar macrophages. Therefore, we exposed WT or Lcn2KO alveolar macrophages to *M.tuberculosis* and measured G-CSF and KC levels in culture supernatants. We show that in alveolar macrophages, *M.tuberculosis*-induced G-CSF and KC production is dependent on endogenous production of Lcn2, since alveolar macrophages derived from Lcn2KO mice induced significantly lower levels of these molecules. In addition, exogenous Lcn2 was able to rescue induction of both these proteins in alveolar macrophages (Figure 4f & 4g). These data suggest that *M.tuberculosis*-induced G-CSF and KC production in alveolar macrophages is partly dependent on endogenous Lcn2 production, but that Lcn2 made by other cells, such as epithelial cells and fibroblasts (Figure 1d), may amplify the response.

### Lcn2 Regulates Inflammation and Contributes to Protective Immunity Against Acute *M.bovis* BCG Infection

**Figure 5:**
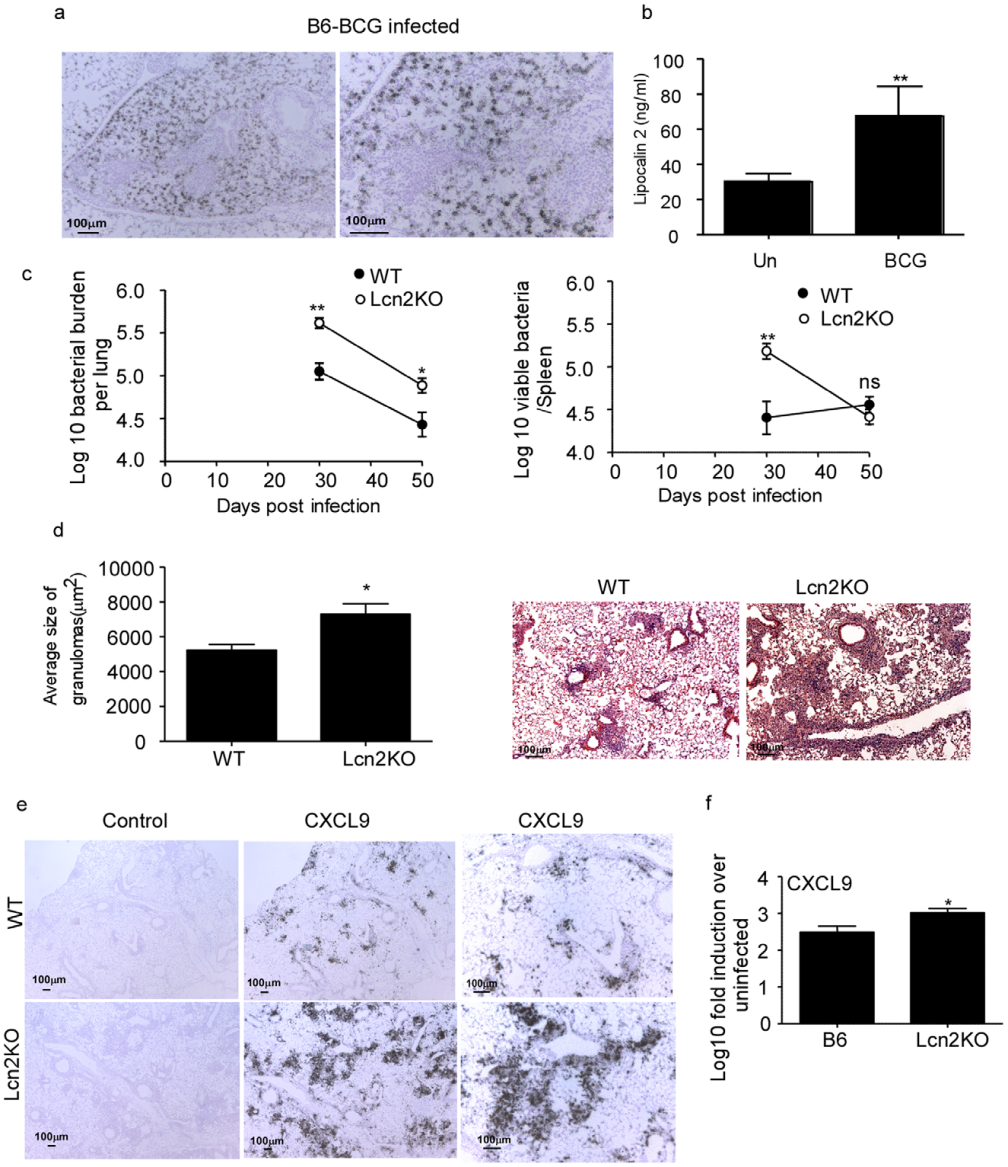
Lcn2 regulates the *M.bovis* BCG induced inflammation and protection in murine lung. Wild type (WT) mice were infected with 5×10^6^ CFU of *M.bovis* BCG by the intratracheal route and day 30 post-infection, formalin-fixed, paraffin-embedded lung sections were assayed for Lcn2 mRNA localization by ISH using a murine Lcn2 mRNA probe (a) and Lcn2 protein levels determined by ELISA (b). Bacterial burden was determined in the lungs and spleen of *M.bovis* BCG infected WT and Lcn2KO mice at the described time points (c). Formalin-fixed paraffin-embedded lungs were stained using H&E and morphometric analysis of the area occupied by the granuloma in H&E stained sections of *M.bovis* BCG infected WT and Lcn2KO lungs was determined (d). A representative section of the lung granuloma is shown at 100X. Formalin-fixed, paraffin-embedded lung sections from infected WT and Lcn2KO were assayed for CXCL9 mRNA localization by ISH using a murine CXCL9 mRNA probe or control probe (e) and total lung CXCL9 mRNA levels induced quantitated using RT-PCR in day 30 infected lungs (f). Original magnification 40X-left panel, middle panel; 100X-right panel (e).The data points represent the mean and SD for four mice for each time point and one experiment representative of two shown. _*_, *p* ≤0.05._ **_, *p* ≤0.005. ns-not significant.

Lcn2 is induced early in the lung in response to *M.bovis* BCG infection from day 2 post infection [18]. Consistent with this data, we found induction of Lcn2 mRNA (Figure 5a) and protein (Figure 5b) in the lungs on day 30 post *M.bovis* BCG pulmonary infection. In addition, we found that Lcn2 mRNA induction in the *M.bovis* BCG-infected lungs were not dependent on IL-23, IL-17 or IL-22 (data not shown). Furthermore, we could detect Lcn2 mRNA within both the lung parenchyma and airways of *M.bovis* BCG infected WT mice (Figure 5a). Although Lcn2 is shown to inhibit intercellular *M.bovis* BCG growth *in vitro* in alveolar epithelial cells [18], it is not known if absence of Lcn2 results in increased susceptibility *in vivo* following pulmonary infection. We found that following pulmonary infection with *M.bovis* BCG, Lcn2KO mice showed increased early bacterial burden that was maintained at later time points (Figure 5c). Interestingly, increased bacterial burden was observed in the spleen at early time points, but was controlled by day 50 post infection (Figure 5c). Importantly, we found that increased lung bacterial burden coincided with increased lung inflammation (Figure 5d). Consistent with our observations in the low dose aerosol model of *M.tuberculosis* infection, we found that lungs of *M.bovis* BCG-infected Lcn2KO mice exhibited increased induction of CXCL9 mRNA when compared to WT *M.bovis* BCG-infected lungs (Figure 5e,f). These data for the first time show that Lcn2 restrains inflammation and is required to mediate immunity to pulmonary acute *M.bovis* BCG infection *in vivo*.

## Discussion

A protective role for Lcn2 in acute high dose *M.tuberculosis* infection has been recently reported [18]. However, given that the natural course of *M.tuberculosis* infection occurs as a result of inhalation through aerosol droplets harboring the bacteria, it is critical to test the protective role of Lcn2 in the low dose aerosol model of *M.tuberculosis* infection. Our results using a low dose aerosol *M.tuberculosis* infection model show that Lcn2 is dispensable for overall protective immunity against primary *M.tuberculosis* infection. Despite a number of studies showing *in vitro* inhibition of *M.tuberculosis* by Lcn2 [17], [18], [19], our data suggests that absence of Lcn2 does not impact overall control of bacteria in the lung or dissemination to other organs following low dose pulmonary *M.tuberculosis* infection in mice. This could be due to a limited role of Lcn2 in inhibiting *M.tuberculosis* growth in some cell types, but not others, and therefore not having an effect on overall bacterial control in the lung in response to chronic *M.tuberculosis* infection. Importantly, we document a new role for Lcn2 in constraining and limiting lymphocytic inflammation and promoting neutrophilic accumulation during mycobacterial infections.

We recently showed that key inflammatory immune mediators following low dose *M.tuberculosis* infection are upregulated in the lung between D12 and D20 post infection [28]. Consistent with this finding, we show here that induction of Lcn2 mRNA also takes place within this crucial time period. Strikingly, our data suggests that early *in vivo* cellular sources of Lcn2 following low dose *M.tuberculosis* infection are within the lung parenchyma, while later sources of Lcn2 are likely both cells within the lung parenchyma and airways. Furthermore, other studies have shown that exposure to *M.tuberculosis* in macrophage cell lines [19], *M.avium* in bone marrow derived macrophages [32] and *M.bovis* BCG in alveolar macrophages [18] induces Lcn2 mRNA, suggesting that pathogenic, non-pathogenic and attenuated strains of mycobacteria can all induce Lcn2 in the host. Furthermore, cytokines such as IL-17 [26], [33] and IL-22 [26] can drive the induction of Lcn2 in epithelial cells. While they are sufficient, they do not appear to be necessary, suggesting that other cytokines can also likely induce Lcn2 during *M.tuberculosis* infection. Furthermore, since IL-23KO *M.tuberculosis*-infected lungs have reduced levels of both IL-17 and IL-22 mRNA [25], this suggests that Lcn2 can be induced even in the absence of both IL-17 and IL-22. Accordingly, other studies have shown that IL-1βinduced in response to extracellular bacterial pneumonia [21], plays a prominent role in infection-induced Lcn2 production, suggesting that it is likely that multiple cytokines can induce Lcn2 expression in response to inflammation.

The new role for Lcn2 presented in this paper is to constrain CXCL9 induction and lymphocytic accumulation during mycobacterial pulmonary infections. Accordingly, Lcn2KO mice exhibit increased granulomatous inflammation, increased lymphocytic accumulation and enhanced CXCL9 mRNA induction in mycobacteria infected lungs. In contrast, Lcn2 also has a pro-inflammatory role by its ability to promote G-CSF and KC production in alveolar macrophages and neutrophil recruitment to the mycobacterial-infected lung. There is precedence for this idea, as a recent study also showed that Lcn2KO mice have reduced neutrophilic accumulation in response to intranasal *Klebsiella* infection [34]. However, despite the dysregulated inflammation found in Lcn2KO *M.tuberculosis*-infected mice, these mice are still able to mediate effective Th1 mediated immunity, induction of anti-mycobacterial killing mechanisms such as induction of iNOS and mediate control of *M.tuberculosis*. We [28], and others [2], have shown that depletion of neutrophils does not impact overall protective outcomes during TB, suggesting that in the presence of an effective Th1 immune responses, efficient neutrophil recruitment is not necessary to mediate immunity against TB. In contrast, a high dose acute *M.tuberculosis* infection model showed that Lcn2KO mice exhibited increased bacterial burden and succumbed to *M.tuberculosis* infection between 6–8 weeks post infection [18]. It is possible that in the high dose model, delivery of high numbers of *M.tuberculosis* by intratracheal route induces more robust production of Lcn2 and that the Lcn2KO mice die due to pathological responses mediated by the dysregulated inflammation, rather than due to just the higher bacterial burden in the lungs of Lcn2KO mice [18]. Consistent with this hypothesis, Lcn2KO mice infected with high dose *M.tuberculosis* intratracheally also showed higher inflammation, which was speculated to be due to the higher bacterial burden [18]. However, since we report increased inflammation and T cell accumulation even under conditions of similar bacterial burden seen in the lungs of Lcn2KO mice in the low dose *M.tuberculosis* infection model, we propose a new physiological role for Lcn2 in regulating inflammation during mycobacterial infections. Based on our data, we suggest that Lcn2 will play a crucial protective role in models of mucosal extracellular bacterial infection where neutrophils recruitment is important for clearance of the pathogen. Accordingly, it has been shown that Lcn2KO mice are more susceptible to mucosal infection with *Klebsiella pneumonia* [21], [34] and *E. coli* pneumonia [8]. In these models it is likely that the Lcn2KO mice are more susceptible not only due to the lack of host iron sequestering by Lcn2, but also because Lcn2 mediates cellular recruitment and regulates inflammation. Lcn2 produced by alveolar epithelial cells has been shown to be involved in intracellular *M.bovis* BCG control [18]. Consistent with this role for Lcn2, using an acute pulmonary *M.bovis* BCG infection model, we show that Lcn2KO mice show increased mycobacterial burden in the lung and peripheral organs. The likely reasons why Lcn2 is required to mediate protective immunity in the *M.bovis* BCG acute pulmonary model but not the low dose *M.tuberculosis* infection model are twofold. Firstly, although Lcn2 has been shown to inhibit both *M.tuberculosis* and *M.bovis* BCG growth in vitro and in cultured cells [17], [18] [18], [19], it is possible that this function of Lcn2 is redundant in vivo during M.tuberculosis infection, but is critical and non-redundant during in vivo *M.bovis* BCG infection. The second, more likely reason for a protective role for Lcn2 in acute pulmonary *M.bovis* BCG infection may reflect its role in chemokine regulation and neutrophil recruitment. This is supported by studies showing that depletion of neutrophils does not impact overall protective immune responses to *M.tuberculosis* infection [28], [35], but results in increased susceptibility to *M.bovis* BCG acute infection [36]. These data together suggest that in acute mycobacterial infection models, Lcn2 regulates inflammation and controls bacterial growth likely through a role in neutrophil recruitment, while in chronic mycobacterial infection models although Lcn2 regulates inflammation, it does not confer protective immunity.

In summary, we project a new role for Lcn2 in pulmonary infections where apart from its role in sequestering iron and inhibiting growth of bacteria, infection-induced Lcn2 can also act on mucosal cells to regulate chemokines such as CXCL9 and restrain inflammation at mucosal sites.
